# Trait-stratified genome-wide association study identifies novel and diverse genetic associations with serologic and cytokine phenotypes in systemic lupus erythematosus

**DOI:** 10.1186/ar3101

**Published:** 2010-07-26

**Authors:** Silvia N Kariuki, Beverly S Franek, Akaash A Kumar, Jasmine Arrington, Rachel A Mikolaitis, Tammy O Utset, Meenakshi Jolly, Mary K Crow, Andrew D Skol, Timothy B Niewold

**Affiliations:** 1 University of Chicago Pritzker School of Medicine, Section of Rheumatology and Gwen Knapp Center for Lupus and Immunology Research, 924 E. 57th St., Chicago, IL, 60637, USA; 2 Rush University, Section of Rheumatology, 1611 West Harrison St., Suite 510, Chicago, IL, 60612, USA; 3 Hospital for Special Surgery, Mary Kirkland Center for Lupus Research, 535 E 70th St., New York, NY, 10021, USA; 4 University of Chicago Pritzker School of Medicine, Section of Genetic Medicine, 5841 S. Maryland Ave., Chicago, IL, 60637, USA

## Abstract

**Introduction:**

Systemic lupus erythematosus (SLE) is a highly heterogeneous disorder, characterized by differences in autoantibody profile, serum cytokines, and clinical manifestations. SLE-associated autoantibodies and high serum interferon alpha (IFN-α) are important heritable phenotypes in SLE which are correlated with each other, and play a role in disease pathogenesis. These two heritable risk factors are shared between ancestral backgrounds. The aim of the study was to detect genetic factors associated with autoantibody profiles and serum IFN-α in SLE.

**Methods:**

We undertook a case-case genome-wide association study of SLE patients stratified by ancestry and extremes of phenotype in serology and serum IFN-α. Single nucleotide polymorphisms (SNPs) in seven loci were selected for follow-up in a large independent cohort of 538 SLE patients and 522 controls using a multi-step screening approach based on novel metrics and expert database review. The seven loci were: leucine-rich repeat containing 20 (*LRRC20*); protein phosphatase 1 H (*PPM1H*); lysophosphatidic acid receptor 1 (*LPAR1*); ankyrin repeat and sterile alpha motif domain 1A (*ANKS1A*); protein tyrosine phosphatase, receptor type M (*PTPRM*); ephrin A5 (*EFNA5*); and V-set and immunoglobulin domain containing 2 (*VSIG2*).

**Results:**

SNPs in the *LRRC20*, *PPM1H*, *LPAR1*, *ANKS1A*, and *VSIG2 *loci each demonstrated strong association with a particular serologic profile (all odds ratios > 2.2 and *P *< 3.5 × 10^-4^). Each of these serologic profiles was associated with increased serum IFN-α. SNPs in both *PTPRM *and *LRRC20 *were associated with increased serum IFN-α independent of serologic profile (*P *= 2.2 × 10^-6 ^and *P *= 2.6 × 10^-3 ^respectively). None of the SNPs were strongly associated with SLE in case-control analysis, suggesting that the major impact of these variants will be upon subphenotypes in SLE.

**Conclusions:**

This study demonstrates the power of using serologic and cytokine subphenotypes to elucidate genetic factors involved in complex autoimmune disease. The distinct associations observed emphasize the heterogeneity of molecular pathogenesis in SLE, and the need for stratification by subphenotypes in genetic studies. We hypothesize that these genetic variants play a role in disease manifestations and severity in SLE.

## Introduction

Systemic lupus erythematosus ((SLE) OMIM #152700) is a complex disease characterized by multi-system involvement commonly affecting the skin, renal, musculoskeletal, and hematopoetic systems. SLE is caused by interactions between susceptibility genes and environmental factors resulting in an irreversible loss of immunologic self-tolerance [1]. Incidence is highest in women during the reproductive years [2]; however, people of all ages, genders, and ancestral backgrounds are susceptible. Disease features range from typically reversible manifestations such as rash or inflammatory arthritis to life-threatening end-organ damage such as glomerulonephritis or thrombosis, and it is difficult to predict which manifestations will affect an individual patient.

Interferon alpha (IFN-α) is a pleiotropic type I interferon with the potential to break immunologic self-tolerance by activating antigen-presenting cells after uptake of self material [3]. Serum IFN-α is elevated in many SLE patients, and elevations often correlate with disease activity [4,5]. Recombinant human IFN-α administered as a therapy for chronic viral hepatitis and malignancy is thought to cause *de novo *SLE in some patients [6]. IFN-α-induced SLE typically resolves after the IFN-α is discontinued [7,8], supporting the idea that IFN-α was causal. We have previously shown that serum IFN-α is abnormally high in 20% of healthy first degree relatives of SLE patients as compared to < 5% of healthy unrelated individuals [9]. Spouses of SLE patients did not have high serum IFN-α. Taken together, these data suggest that high serum IFN-α is a heritable risk factor for SLE. Additionally, serum IFN-α activity is highest during the ages of peak SLE incidence in both patients and their healthy first degree relatives [10]. The high IFN-α trait in SLE families is inherited in a complex fashion, suggesting polygenic inheritance which has not been fully characterized [11].

Autoantibodies directed at double-stranded DNA (dsDNA) and RNA binding proteins (anti-Ro, anti-La, anti-Sm, and anti-RNP, collectively anti-RBP) are characteristically found in SLE sera, and are the strongest known predictors of high serum IFN-α in SLE patients [9]. The presence of anti-RBP and anti-dsDNA autoantibodies appears to be heritable in SLE families [12]. These autoantibodies are rare in healthy first degree relatives (one to three percent prevalence), and thus heritability of autoantibody traits is not sufficient to explain the heritability of high serum IFN-α in SLE families [9]. Immune complexes formed by these SLE-associated autoantibodies can directly stimulate IFN-α production *in vitro*, likely via the endosomal Toll-like receptors [13]. In humans *in vivo*, we find that while anti-RBP antibodies are associated with high serum IFN-α, they are not sufficient [14]. This suggests that although these SLE-associated autoantibodies are correlated and likely mechanistically related to serum IFN-α, other factors such as genetic variation will likely be important to *in vivo *IFN-α levels.

We have begun to genetically map the high IFN-α trait in SLE, and found that a number of genetic variants which are associated with susceptibility to SLE are also associated with increased serum IFN-α, including variants of interferon regulatory factor 5 (*IRF5*) [15], interferon regulatory factor 7 (*IRF7*) [16], protein tyrosine phosphatase non receptor type 22 (*PTPN22*) [17], and osteopontin (*SPP1*) [18]. We have also shown that the autoimmune disease associated variant of signal transducer and activator of transcription 4 (*STAT4*) modulates cellular sensitivity to IFN-α signaling [19]. These studies provide further support for the hypothesis that IFN-α pathway dysregulation is a primary causal factor in human SLE. This work also suggests that the serum IFN-α trait could be a useful tool in identifying additional genetic loci related to SLE pathogenesis.

In this study, we performed a genome-wide association study (GWAS) on 104 SLE patients stratified by extremes of phenotype in serum IFN-α. Given the importance of anti-RBP and anti-dsDNA antibodies on serum IFN-α, patients were also stratified by the presence or absence of these antibodies. The size of the GWAS study did not allow for rigorous exclusion of false positives, so we used a multi-step screening algorithm which included novel metrics derived from the GWAS chip data and expert literature and database review to increase the likelihood of selecting *true positive *SNPs for validation. SNPs selected were then genotyped in an independent SLE cohort, testing for association with autoantibody profile and serum IFN-α in a case-case study design, as well as overall association with SLE in a case-control study. This approach was chosen to maximize our opportunity for novel gene discovery in a moderate-sized well-characterized cohort with complete molecular phenotype data. The trait-stratified GWAS screening approach followed by selected validation was successful, and we report seven novel loci which are strongly associated with autoantibodies and serum IFN-α, two important heritable subphenotypes in SLE.

## Materials and methods

### Patients and samples

The initial cohort of SLE patients studied in the GWAS scan was obtained from the Hospital for Special Surgery Lupus Registry, and consisted of 104 SLE patients, including 20 African-American, 36 European-American, 32 Hispanic-American, and 16 Asian-American SLE patients. Patients were selected from the top 33% and bottom 33% of serum IFN-α activity, and were additionally stratified by ancestry and the presence or absence of anti-RBP or anti-dsDNA antibodies. The independent validation cohort of 538 SLE patients was obtained from the University of Chicago Translational Research in the Department of Medicine (TRIDOM) registry and Rush University Medical Center, and consisted of 280 African-American, 173 European-American, and 85 Hispanic-American SLE patients. The validation cohort was not selected for inclusion in the study based upon any particular SLE-associated phenotype. All patients met the revised 1982 American College of Rheumatology criteria for the diagnosis of SLE [20]. Samples from 522 controls were obtained from the TRIDOM registry, including 361 African-American and 161 European-American subjects who were individually screened for the absence of autoimmune disease by medical record review and matched by sex to the cases. The controls were on average older than the cases, although given our ability to screen for the absence of autoimmune disease this should be of benefit as one would assume that older individuals who do not have autoimmune disease are likely a low-risk group for autoimmunity. Clinical characteristics and demographic information for the patients and controls in the validation cohort are summarized in Supplemental table S1 in Additional file 1. Informed consent was obtained from all subjects at each site, and the study was approved by the institutional review board at each institution.

### Measurement of serum IFN-α activity and autoantibodies

The reporter cell assay for IFN-α has been described in detail elsewhere [9,21]. In brief, reporter cells (WISH cells, ATCC #CCL-25) were used to measure the ability of patient sera to cause IFN-induced gene expression. The output of the assay is interpreted as a functional activity of IFN-α in the serum sample. Results from the IFN-α assay were standardized to a healthy multi-ancestral reference population as previously described, and a serum IFN-α activity score was calculated based upon the mean and SD of the reference population [9]. Antibodies to anti-Ro, anti-La, anti-Sm, and anti-RNP were measured in all samples by ELISA methods, and anti-dsDNA antibodies were measured using *Crithidia luciliae *immunofluorescence, with detectable fluorescence considered positive. Autoantibodies were assayed in the TRIDOM and Rush University samples in the University of Chicago clinical laboratory, and the Hospital for Special Surgery samples used in the GWAS screening were assayed in the Hospital for Special Surgery clinical laboratory. Standard clinical lab cutoff points were used to categorize samples as positive or negative. There were no significant differences in autoantibody prevalence between the two clinical laboratories.

### Screening GWAS study

The initial cohort of SLE patients studied in the GWAS scan consisted of 104 SLE patients obtained from the Hospital for Special Surgery Registry. A study design incorporating multiple ancestral backgrounds was chosen, as both autoantibodies and serum IFN-α levels are heritable pathogenic factors which are shared between all ancestral backgrounds [9,12]. This does not ensure that the same factors will underlie the serum IFN-α trait in all backgrounds, and we considered each ancestral background separately in the GWAS. Patients were selected in an "extremes of phenotype" design from the top 33% and bottom 33% of serum IFN-α activity, and were additionally stratified by ancestry and the presence or absence of anti-RBP or anti-dsDNA antibodies. Genomic DNA from SLE patients in the HSS registry was combined into pools of four individuals matched by ancestry, IFN-α levels, and presence or absence of anti-RBP or anti-dsDNA autoantibodies, respectively. The high IFN-α group was defined as those patients in the top 33% of serum IFN-α who had one or more of the following autoantibody specificities: Ro, La, Sm, RNP, or dsDNA, and the low group was defined as those in the bottom 33% of serum IFN-α who lacked these autoantibodies. These pools containing equal amounts of DNA from four individuals matched by these traits were run on Affymetrix 500K SNP mapping chips (Santa Clara, CA, USA) per manufacturer protocol. Data from the individual probes were then analyzed, and probe intensity was used to represent allelic frequencies present in the pooled DNA. Probe data were summarized and analyzed using Gene Pool by Pearson JV *et al*. [22]. We considered two metrics to help identify allele frequency differences between the high vs. low IFN-α groups and the present vs. absent anti-RBP or anti-dsDNA groups. The first metric is the Silhouette score proposed by Pearson *et al*. [22].

We also developed our own score which is the difference in the weighted mean of the relative allele signals (RAS = intensity of A allele/(intensity of A + intensity of B allele)) for each ethnic group (EA, AA, HI). The weights were constructed as the inverse standard deviation of the RAS score calculated from the 8 (or 12) probes per SNP present on the Affymetrix array. The weights were then normalized so that they sum to one. Our adjusted RAS score is then calculated by summing each group's weighted RAS score. The goal of constructing our own statistic was three-fold: First, it down-weighted SNPs that had highly variable signals even if the mean differences between groups was large; this allows a certain measure of quality control that is not offered as effectively by the Silhouette score; second, it accounts for heterogeneity of allele frequencies among the ancestral groups; third, it creates a statistic that is robust to population stratification which can lead to false positives.

Specifically, the weighted mean RAS score is calculated as follows:

Define r_i,j,k _= RAS score for the j^th ^control (i = 0) or case (i = 1) of ethnicity k = {EA, AA, Asian, HA}, v_i,j,k _= variance of the i,j,k^th ^RAS score.

$$r_{i,k}=\frac{\sum_{j=1}^{{\text{n}}_{\text{i,k}}}r_{i,j,k}}{\sqrt{v_{i,j,k}}}$$

$$v_{i,k}={\sum_{j=1}^{{\text{n}}_{\text{i,k}}}\left(\sqrt{v_{i,j,k}}/{\text{n}}_{\text{i,k}}\right)}^{2}$$

$$Z=\sum_{\text{k}=1}^{3}\left(r_{\text{1,k}}-r_{\text{0,k}}\right)/\sqrt{v_{\text{0,k}}+v_{1,\text{k}}}$$

Of the 104 subjects studied in the GWAS, 44 were in the high IFN-α/autoantibody positive group and 60 were in the low IFN-α/autoantibody negative group. It was difficult to match even numbers of subjects between these two groups in each ancestral background due to the stringent double phenotype case selection criteria, and some of the ancestral groups deviated from a 1:1 match (proportions by ancestral background were as follows: Asian-American 8 high and 8 low, African-American 12 high and 8 low, European-American 12 high and 24 low, Hispanic-American 12 high and 20 low). This was not confounding, as the data from each ancestral background were analyzed separately.

### Selection of SNPs from GWAS study for validation

We next employed a multiple step screening algorithm to select a subset of SNPs for replication that were highly suggestive based on both their Silhouette and weighted RAS scores. SNPs were first ordered by weighted RAS score, and the top 200 SNPs by RAS score were considered. SNPs on this list with a Silhouette score that was not within the top 200 Silhouette scores were removed (31%), leaving SNPs that ranked in the top 200 by both metrics. These remaining SNPs were again ranked by weighted RAS score, and the location of each of the top 50 SNPs was examined in detail (Supplemental table S2 in Additional file 1). Similar to other autoimmune diseases, SNPs which are associated with SLE in unbiased genome-wide mapping studies are largely within or near genes with immune system function [23], and we presumed that our study design which stratified patients by two immune system phenotypes would likely yield SNPs in genes related to the immune system. Therefore, we assumed that the prior probability of a true association was increased if 1) the SNP was located within or near a known gene, and 2) if that locus had immune system relevance based upon public databases. Nine SNPs of the top 50 met both of these criteria (Supplementary table S2 in Additional file 1).

### SNP genotyping in the validation cohort

Individuals in the validation cohort were genotyped at the following SNPs using ABI Taqman Assays-by-Design primers and probes: rs10762360 (leucine-rich repeat containing 20, *LRRC20*), rs10784318 (protein phosphatase 1 H, *PPM1H*), rs10980684 (lysophosphatidic acid receptor 1, *LPAR1*), rs2820223 (ankyrin repeat and sterile alpha motif domain containing 1A, *ANKS1A*), rs17494870 (protein tyrosine phosphatase, receptor type M, *PTPRM*), rs26725 (ephrin A5, *EFNA5*), and rs112219769 (V-set and immunoglobulin domain containing 2, *VSIG2*). Scatter plots were all reviewed individually for quality, and none of the SNPs genotyped deviated significantly from the expected Hardy-Weinberg proportions (*P *> 0.01 for all SNPs in all ancestral backgrounds). Each SNP assay resulted in successful genotype calls in > 98% of the subjects, and none of the cases or controls were excluded due to incomplete or unsuccessful genotyping.

### Statistical analysis in validation cohort

Logistic regression models were used to detect associations between the presence of autoantibodies and genotype at candidate SNPs. We performed this analysis because the initial GWAS study was stratified by both serum IFN-α and autoantibodies, and we sought to detect and clarify any potential associations with serology. Screening models incorporated each of the SNPs as predictor variables with a single autoantibody trait as the outcome variable in each ancestral background separately, and backward step-wise regression was performed discarding predictors which did not show evidence for association (*P *> 0.1). When more than one SNP was significantly associated with a particular antibody, interaction terms were included to detect potential SNP-SNP interactions. SLE-associated autoantibodies can show associations with other autoantibodies, (for example, the presence of anti-Sm is highly correlated with the presence of anti-RNP) and so we undertook additional logistic regression modeling to explore and refine SNP-antibody associations if one allele of a SNP was associated more strongly with a combination of antibodies than with either antibody alone. To test combination models, we generated new outcome variables which represented a combination of the two associated autoantibodies based upon the direction of the associations observed in the original regressions (such as presence of both anti-Ro and anti-dsDNA vs. the absence of one or both of those antibodies). If the antibody combination outcome variable provided significantly improved fit as compared to the individual autoantibody outcome variables, then the combination was reported as the best fit model.

To account for potential differences in admixture or population structure within self-reported ancestral backgrounds in the validation cohort, we performed a principal component analysis which included 13 independent SNPs (including the seven described in this study) that had been genotyped in all of our cases and controls. The first component obtained in this analysis provided a strong separation between subjects of self-reported African-American ancestry and self-reported European-American ancestry. Using the central value for this component to categorically split the population into African-derived vs. European-derived ancestry bins, self-reported African-American subjects were concordant with the African-derived ancestry bin 75.1% of the time, and self-reported European-Americans were concordant with the European-derived ancestry bin 86.3% of the time (*P*-value for a random distribution of self-reported ancestral backgrounds between these two bins = 1.3 × 10^-91^). Self-reported Hispanic-American ancestry subjects were largely overlapping with the European-American subjects in this analysis. We retested our autoantibody regression models and case-control analyses incorporating the first principal component as a quantitative covariate designating proportional European- vs. African-derived ancestry, and did not find significant differences in autoantibody associations with or without the inclusion of the first principal component in the regression (data not shown). The lack of a significant impact upon the reported associations provides some reassurance that these associations are not the result of differences between cases and controls in proportional admixture or population structure differences within self-reported ancestral backgrounds.

To analyze serum IFN-levels in the context of SNP genotype, column graphs were constructed to compare quantitative IFN-α levels between genotype categories. Because IFN-α levels were far from normally distributed, we used a non-parametric version of the two sample t-test (non-parametric Mann-Whitney U) to test if IFN-α levels varied significantly between two SNP genotype groups. Case-control allele frequency differences were tested using a chi-square test of independence. Reported *P*-values are not adjusted for multiple comparisons.

## Results

### Results of GWAS screening study and multi-step screening algorithm

The top 50 SNPs from the screening GWAS study were determined using a combination of their Silhouette and weighted RAS scores (see Methods, Supplemental table S2 in Additional file 1), and we examined each of these SNPs in detail. When a SNP was located within or near a gene, a comprehensive literature and public database search was performed on each gene. We assumed that the prior probability of a true association was increased if, 1) the SNP was located within or near a known gene, and 2) if that locus had immune system relevance based upon public databases. Nine SNPs located in or near the following eight genes met both criteria: *STAT4, LRRC20, PPM1 H, LPAR1, ANKS1A, PTPRM, EFNA5*, and *VSIG2*. Two SNPs in the top 50 were located in the *ANKS1A *region, and in this case the SNP that was statistically stronger in the GWAS study was chosen for validation. The SNP in *STAT4 *(rs9967792) was located in the third intron of the gene. This SNP is in the LD block immediately adjacent to the SLE-associated haplotype block of *STAT4 *[24,25]. We have previously demonstrated that variation in this third intron of *STAT4 *was associated with both serum IFN-α and autoantibody profile in multiple ancestral backgrounds in a study which included some of the same individuals [19], and we presumed that this was a recapitulation of that result, and did not pursue this SNP. Finding *STAT4 *in the top 50 SNPs demonstrated that *true positive *SNPs were indeed included among the top 50 in our screening GWAS study. Statistical results from the GWAS in each ancestral background separately for the seven SNPs chosen for validation are shown in Supplemental table S3 in Additional file 1.

### Association of GWAS candidates with serologic subsets in SLE

Individuals in the validation cohort were genotyped at the following SNPs: rs10762360 (*LRRC20*), rs10784318 (*PPM1H*), rs10980684 (*LPAR1*), rs2820223 (*ANKS1A*), rs17494870 (*PTPRM*), rs26725 (*EFNA5*), and rs112219769 (*VSIG2*). We used logistic regression models to test candidate SNPs from the initial GWAS for potential serologic associations in the validation cohort. SLE-associated autoantibodies can show associations with other autoantibodies, and so if one SNP was associated with more than one antibody we undertook additional logistic regression modeling to explore genetic associations with antibody combinations (see Methods). Output from the initial screening and follow up models is reported for each ancestral background separately in Supplemental tables S4-6 in Additional file 1. These regressions identified strong serologic associations for five of the seven SNPs which were surprisingly diverse, both in the specific associated autoantibody profile as well as the particular ancestral background (Table 1). Many of these serologic associations were very strong with OR > 2.0, and five of the seven SNPs had *P-*values which exceeded a Bonferroni threshold of 1.4 × 10^-3^, allowing for correction for both the number of SNPs tested and the number of traits considered. No SNP-SNP interactions were detected. Alleles of the SNPs in both *LPAR1 *and *ANKS1A *were associated with more than one autoantibody in the initial regressions, and follow-up regression analysis supported a best-fit model in which these alleles were associated with the presence of the two autoantibodies simultaneously. The *VSIG2 *rs112219769 T allele showed a positive association with anti-RNP antibodies and an inverse association with anti-Sm antibodies. This was surprising, as the presence of anti-Sm autoantibodies is correlated with the presence of anti-RNP antibodies. The best-fit model for the *VSIG2 *SNP was achieved with the antibody profile *anti-RNP positive, anti-Sm negative*.

**Table 1:** Serologic associations observed in SLE patients in the replication cohort, best fit model from logistic regression

Locus and Allele	Associated Antibody Profile	Ancestral Background	Odds Ratio (95% CI)	*P*-value
LRRC20 rs10762360 G	Anti-La	AA	2.40 (1.51 to 3.81)	1.6 × 10^-4^
PPM1 H rs10784318 C	Anti-La	EA and HA joint	3.74 (1.88 to 7.47)	3.6 × 10^-5^
LPAR1 rs10980684 T	Anti-Ro with Anti-Sm	AA and HA joint	2.22 (1.42 to 3.47)	1.2 × 10^-4^
ANKS1A rs2820223 C	Anti-Ro with Anti-dsDNA	AA	3.14 (1.68 to 5.87)	3.5 × 10^-4^
PTPRM rs17494870 C	-	-	-	-
EFNA5 rs26725 C	Anti-RNP	AA	1.73 (1.10 to 2.72)	0.018
VSIG2 rs11219769 T	Anti-RNP lacking Anti-Sm	EA and HA joint	3.99 (1.81 to 8.75)	2.1 × 10^-4^

AA, African-American, EA, European-American, HA, Hispanic-American, 95% CI, 95% confidence interval; "joint" indicates that two or more ancestral backgrounds showed a similar tendency when analyzed separately with a non-significant difference observed between odds ratios, and that the indicated ancestral backgrounds were analyzed jointly using a fixed-effects model.

### Association of GWAS candidates with serum IFN-α activity in the SLE cohort

**Figure 1:**
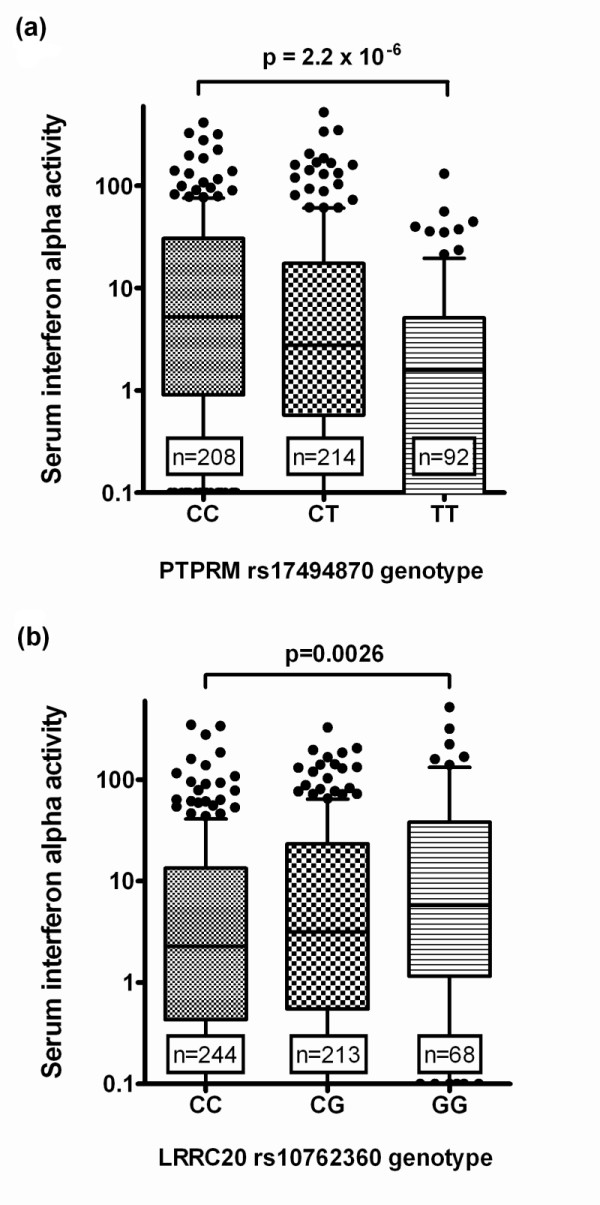
**Serum IFN-α levels in SLE patients stratified by SNP genotype at PTPRM (a) and LRRC20 (b)**. Y-axis shows the serum IFN-α activity score as outlined in the Methods section. Bars show the median, error bars show the interquartile range. Because IFN-α levels were far from normally distributed, we used a non-parametric version of the two sample t-test (non-parametric Mann-Whitney U) to test if IFN-α levels varied significantly between the two homozygous genotype groups.

**Figure 2:**
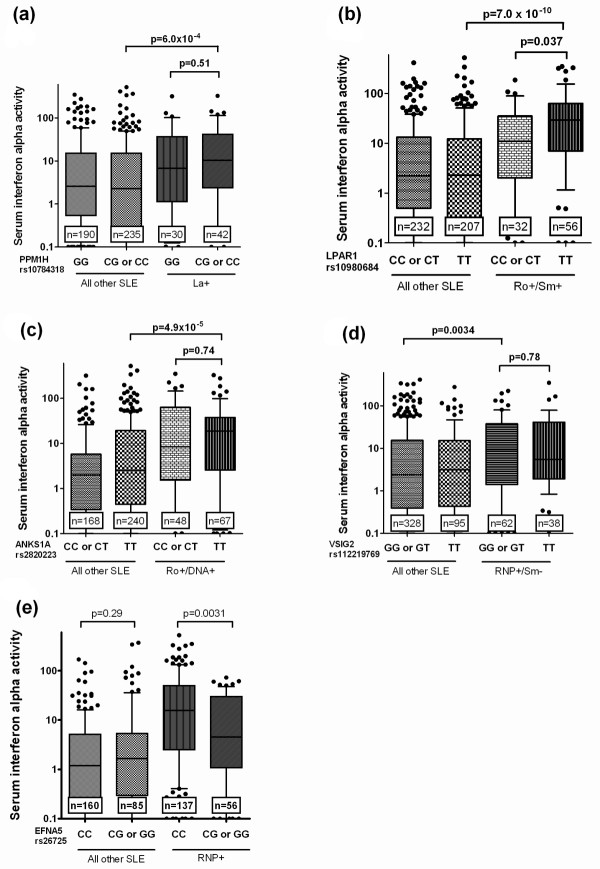
**Serum IFN-α levels in SLE patients stratified by genotype at PPM1 H (a), LPAR1 (b), ANKS1A (c), VSIG2 (d), and EFNA5 (e)**. Minor allele homozygotes are combined with heterozygotes for analysis. Patients are stratified first by genotype, and secondarily by the serologic factor associated with the particular SNP in Table 1, as indicated in the legend below the X-axis. Y-axis shows the serum IFN-α activity score as outlined in the Methods section. Bars show the median, error bars show the interquartile range. *P*-values indicate two column comparisons between the bars on the graph indicated by the line, and are calculated using the Mann-Whitney U test. Significant differences are observed either between subjects with the same genotype but with differing serological profiles (a through d), or between subjects with different genotypes but sharing the same serologic profile (e).

We next tested for relationships between SNP genotype at the candidate SNPs and serum IFN-α in the SLE patients in the validation cohort. Genotype at the SNPs in *PTPRM *and *LRRC20 *showed a statistically significant relationship with serum IFN-α in SLE patients when ancestral backgrounds were analyzed in aggregate (*P *= 2.2 × 10^-6 ^and *P *= 2.6 × 10^-3 ^respectively, both exceeding Bonferroni threshold *P*-value of 7.1 × 10^-3^, Figure 1). The C allele of rs17494870 (*PTPRM*) did not show any evidence for association with serologic profile, however this allele was the most strongly associated with serum IFN-α, and the association was independently observed in multiple ancestral backgrounds (Supplemental figure S1 in Additional file 1). In African-Americans, we had observed an association between the *LRRC20 *rs10762360 G allele and anti-La, but surprisingly we found no association between genotype at this SNP and IFN-α in African-Americans. Instead, there was a highly significant relationship between serum IFN-α and *LRRC20 *genotype in a joint analysis of European- and Hispanic-American subjects (*P *= 2.1 × 10^-4^), in whom the serologic association with anti-La antibodies was not present (Supplemental figure S2 in Additional file 1). The SNPs in *PPM1 H, LPAR1, ANKS1A*, and *VSIG2 *genes did not demonstrate a relationship with serum IFN-α, however the antibody profiles associated with these genes were strongly associated with increased serum IFN-α, suggesting a secondary association (Figure 2). A distinct pattern of association was observed between the SNP in the *EFNA5 *locus and serum IFN-α. The *EFNA5 *rs26725 C allele was associated with increased IFN-α only in those subjects who had anti-RNP antibodies, suggesting a cooperative effect between the rs26725 C allele and anti-RNP antibodies upon serum IFN-α (Figure 2e). While we have previously shown that serum IFN-α is higher in younger SLE patients, none of the SNPs tested were significantly associated with age of the subjects, and thus age should not be driving the differences we observed related to SNP genotype.

**Figure 3:**
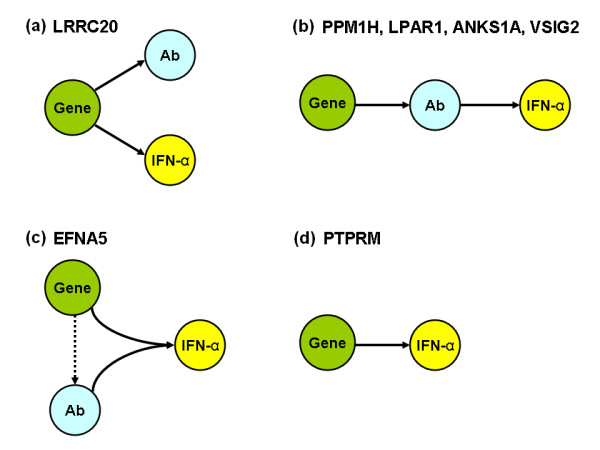
**Diagrams of the patterns of association observed between SNP genotype, serologic profile, and serum IFN-α**. "Gene" represents the genetic variation in the locus studied, and "Ab" represents the particular serologic profile associated with that locus. Connecting lines between nodes indicate associations, and arrowheads show the hypothesized direction of the relationship. The loci demonstrating each pattern are indicated following the letter labeling the panel. The dashed line in C. indicates that a suggestive association was seen between the EFNA5 SNP genotype and the same serologic profile which is linked to IFN-α, but this serologic association did not withstand Bonferroni correction for multiple comparisons.

The different patterns of association between these SNPs and serum IFN-α and autoantibody traits were striking, and allowed us to place pathogenic events in order to some degree (Figure 3). The patterns observed could be summarized into two major categories as they relate to serum IFN-α: one in which the gene exerts an influence on serum IFN-α independent of any autoantibody association (*PTPRM *and *LRRC20*), and a second pattern in which the gene is associated with serum IFN-α secondarily due to an association with an autoantibody profile that is characterized by high IFN-α (*PPM1H*, *ANKS1A*, *LPAR1*, *VSIG2*). A third pattern of cooperative association was observed with *EFNA5*, in which IFN-α was significantly increased only when the particular allele and specific autoantibody are found together. This was similar to the pattern we had observed with the SLE-associated variants of *IRF5 *and *IRF7 *[15,16].

### Case-control Association Study of the Seven Candidate loci from GWAS

There were no significant differences in allele frequencies between SLE cases and non-autoimmune controls in overall case-control analyses in African- and European-American ancestral backgrounds (Supplemental table S7 in Additional file 1). Differences in allele frequencies for each SNP in each ancestral background were modest (most ORs were in the 0.8 to 1.2 range). The size of our case-control populations do not allow us to exclude the possibility that an association exists, but it appears that the most dramatic genetic effects observed with these SNPs in SLE will be upon subphenotypes.

## Discussion

In this study, we used a detailed subphenotyping strategy to detect genetic factors associated with two important heritable phenotypes in SLE: autoantibody traits and serum IFN-α activity. Both of these traits have been associated with increased disease activity in SLE [5,26], and both are thought to play a pathogenic role in disease initiation [9,27]. The number and strength of the genetic associations discovered suggests that this technique could be a powerful tool in other diseases. A version of this study design has been used in rheumatoid arthritis, as patients are often stratified by the presence or absence of anti-cyclic citrullinated peptide antibodies, and large differences in genetic associations between these two serologic subsets have been observed [28]. The seven loci that we found to be strongly associated with subphenotypes in SLE could potentially be discovered in a large case-control study, however these loci would likely be characterized by modest odds ratios for association in a traditional case-control study design based upon our case-control data. A limitation of our study is that we did not have complete clinical data available for all of the cases in the study, and thus we did not attempt to detect associations with clinical features of disease. Another limitation is that we did not have dense ancestry-informative genetic markers available in our cohort, and the pooled design of the GWAS study limited to some degree the amount of ancestry-related genetic information obtained from individual subjects in the GWAS portion of the study. As noted in the Methods, we tested associations within each self-reported ancestral background separately, used statistics that are robust to differences related to ancestry in the GWAS meta-analysis, and also used statistical methods to address potential confounding by differences in proportional ancestry within self-reported ancestral groups in the validation cohort. These analyses did not suggest that our results were confounded by ancestry. Follow-up replication and fine-mapping studies of these seven loci in independent multi-ancestral cohorts with dense ancestry-informative markers would be warranted.

The SNPs examined in this study are located in or near genes which are both novel in their association with SLE and highly interesting as potential immunomodulatory loci. The *LRRC20 *gene product has not been studied to date, but is known to contain leucine-rich repeats which are similar to the Toll-like receptor family of proteins involved in microbial pattern sensing and stimulation of cytokine pathways, including the IFN-α pathway. *PTPRM *is a receptor-type protein tyrosine phosphatase [29], which is of particular interest as a number of protein tyrosine phosphatases have been linked to autoimmune disease, including *PTPN22 *and *PTPN2 *[1,30]. PTPRM mediates cell-cell aggregation via its extracellular domain [29], and the phosphatase domain allows intra-cellular signaling. *PPM1 H *is an intra-cellular phosphatase which is involved in regulation of apoptosis, possibly via the p53 pathway [31]. *LPAR1 *and *EFNA5 *encode receptors expressed on leukocytes which can mediate apoptosis and chemotaxis [32], and leukocyte adhesion [33] respectively. Interestingly, genetic variation 124 kb upstream of *LPAR1 *was recently associated with circulating monocyte count in European populations [34]. *ANKS1A *is also expressed in leukocytes and can be phosphorlyated by Lck [35], suggesting a potential role in T-cell signaling. After completion of our study, a SNP near *ANKS1A *in the immediately adjacent UHRF1 binding protein 1 (*UHRF1BP1*) locus was reported to confer modest risk of SLE (OR = 1.17) in a large case-control study in European ancestry [36]. While we observed the major association between *ANKS1A *and SLE phenotype in African-Americans, it is possible that with a larger European ancestry cohort we may have observed an association between this locus and SLE phenotype, and this genomic region clearly warrants further study in multiple ancestral backgrounds. *VSIG2*, also known as cortical thymocyte receptor like, is expressed in the thymus and may be involved in antigen presentation [37]. There are no known direct physical interactions between these gene products, although functionally each of these genes converge upon important immune system features involved in SLE pathogenesis, such as cell-cell interaction, antigen responses, and apoptosis.

Of the SLE risk loci which have previously been associated with serum IFN-α levels in SLE patients [15-19,38], only *STAT4 *was represented in our top 50 SNP list. It is important to note that our screening GWAS study was not powered to exclude potential associations with serum IFN-α in SLE with any degree of certainty, and absence of a gene from our list does not mean that it cannot be associated with serum IFN-α phenotype in SLE. We were encouraged that one of these previously reported IFN-α-associated loci was present in the top 50 list, supporting the idea that we were able to identify some true positives. We would estimate that many more loci will be associated with the serum IFN-α phenotype in SLE, and this study is by no means exhaustive. It is interesting that the human leukocyte antigen (HLA) locus was not represented in our list of top SNPs, as this locus is known to be one of the strongest associations with SLE and many other autoimmune diseases [23,39]. Different HLA alleles are known to be associated with particular autoantibody profiles in SLE [40]. The HLA locus is highly complex and shows a large degree of variation by ancestry, and it is possible that the multi-ancestral nature of our screening GWAS cohort did not allow detection of HLA associations.

The success of this study design suggests that application of a similar technique in other diseases could be highly advantageous, allowing for robust gene discovery related to important pathways and subphenotypes using a smaller cohort than is typically studied in case-control GWAS. Additionally, we demonstrate a number of different patterns of association between SNP genotype and molecular subphenotypes (Figure 3). These intricate subphenotype relationships begin to suggest pathogenic models, which can be followed up in mechanistic studies. This is another strength of this study design, as more information regarding the potential function and role in disease pathogenesis is immediately available for the candidate genes as compared to standard case-control designs. The models outlined in Figure 3 can be tested functionally, allowing for integration of these loci into the mechanisms of human SLE pathogenesis.

## Conclusions

We compare patients from the extremes of high vs. low interferon and autoantibody levels, and identify a number of novel genetic regions that are associated with high interferon alpha and particular autoantibodies in SLE patients. These genetic associations were very strong in the particular patient groups who demonstrated the specific findings and were not as strong in the overall cohort, suggesting that this approach was necessary to discover these genes. These genotype-phenotype relationships begin to suggest pathogenic models, which can be followed up in mechanistic studies and allow these genes to be integrated into the framework of human SLE pathogenesis. The number and strength of the genetic associations discovered suggests that this technique could be a powerful tool in other diseases. Because both autoantibody profile and serum IFN-α are important heritable pathogenic factors in SLE, we hypothesize that the gene variants we describe will play a role in disease susceptibility and severity in SLE.

## Abbreviations

*ANKS1A*: ankyrin repeat and sterile alpha motif domain containing 1A; ANTI-RBP: anti-RNA-binding protein antibodies; DSDNA: double-stranded DNA; *EFNA5*: ephrin A5; GWAS: genome-wide association study; HLA: human leukocyte antigen; IFN-α: interferon alpha; *IRF5*: interferon regulatory factor 5; *IRF7*: interferon regulatory factor 7; KB: kilobases; *LPAR1*: lysophosphatidic acid receptor 1; *LRRC20*: leucine-rich repeat containing 20; OR: odds ratio; *PPM1H*: protein phosphatase 1H; *PTPN22*: protein tyrosine phosphatase, non-receptor type 22; *PTPRM*: protein tyrosine phosphatase, receptor type, M; RAS: relative allele signals; SLE: systemic lupus erythematosus; SNP: single nucleotide polymorphism; *SPP1*: osteopontin; *STAT4*: signal transducer and activator of transcription 4; TRIDOM: Translational Research in the Department of Medicine; *UHRF1BP1*: UHRF1 binding protein 1; *VSIG2*: V-set and immunoglobulin domain containing 2.

## Competing interests

MKC has a patent pending for the interferon assay used in this paper, and TOU has received consulting fees from Genentech. All other authors declare that they have no competing interests.

## Authors' contributions

SNK participated in genotyping the replication cohort, interferon assays, data analysis and interpretation, and drafted the initial manuscript. BSF participated in genotyping the replication cohort, interferon assays, and data analysis. AAK and JA participated in genotyping the replication cohort and data analysis. RAM, TOU and MJ participated in patient recruitment and data analysis. MKC supported and participated in the planning of the GWAS study and data analysis and interpretation. ADS analyzed the GWAS data, and developed novel methods and metrics for this study. TBN conceived and designed the study, performed the SNP selection algorithm, and supervised all experiments, analysis, and the preparation of the manuscript. All authors participated in the preparation of the manuscript, and approve of the final version.

## Supplementary Material

Additional file 1 **Supplemental material**. All supplementary tables, figures, and figure legends are contained in this document file. Click here for file
