# Supplementary material for: Trait-stratified genome-wide association study identifies novel and diverse genetic associations with serologic and cytokine phenotypes in systemic lupus erythematosus
Source: Arthritis Res Ther. 2010 Jul 26;12(4):R151. doi: 10.1186/ar3101 (PMC2945049; doi:10.1186/ar3101)
Supplement: Additional file 1 — Supplemental material. All supplementary tables, figures, and figure legends are contained in this document file. [file ar3101-S1.DOC]

**Supplemental Table 1** Clinical and serologic characteristics of the validation cohort.

|  | **Clinical Feature** | **Cases** | | | **Controls** | |
| --- | --- | --- | --- | --- | --- | --- |
|  |  | **AA**  **n=280** | **EA n=173** | **His**  **n=85** | **AA**  **n=361** | **EA**  **n=161** |
| **Demographic characteristics** | **Median age (yrs)** | 42 | 44 | 39 | 53 | 49 |
|  | **Interquartile age range (yrs)** | 30-51 | 32-55 | 29-50 | 46-62 | 40-61 |
|  | **% Female** | 0.90 | 0.87 | 0.87 | 0.87 | 0.85 |
| **ACR Clinical Criteria for SLE** | **Malar rash** | 0.50 | 0.56 | 0.60 | - | - |
|  | **Discoid rash** | 0.30 | 0.08 | 0.20 | - | - |
|  | **Photosensitivity** | 0.22 | 0.38 | 0.27 | - | - |
|  | **Oral ulcers** | 0.33 | 0.28 | 0.47 | - | - |
|  | **Arthritis** | 0.82 | 0.82 | 0.83 | - | - |
|  | **Serositis** | 0.35 | 0.37 | 0.57 | - | - |
|  | **Renal disorder** | 0.38 | 0.29 | 0.30 | - | - |
|  | **Neurological disorder** | 0.18 | 0.14 | 0.07 | - | - |
|  | **Hematological disorder** | 0.69 | 0.63 | 0.47 | - | - |
|  | **Immunological disorder** | 0.89 | 0.88 | 0.87 | - | - |
|  | **ANA** | 0.99 | 0.98 | 1.00 | - | - |
| **Prevalence of Specific Autoantibody Profiles** | **Ro** | 0.51 | 0.35 | 0.38 | - | - |
|  | **La** | 0.19 | 0.11 | 0.09 | - | - |
|  | **Sm** | 0.38 | 0.12 | 0.25 | - | - |
|  | **RNP** | 0.63 | 0.19 | 0.35 | - | - |
|  | **DNA** | 0.38 | 0.42 | 0.45 | - | - |
|  | **Ro with Sm** | 0.23 | 0.05 | 0.15 | - | - |
|  | **Ro with DNA** | 0.23 | 0.17 | 0.20 | - | - |
|  | **RNP lacking Sm** | 0.27 | 0.10 | 0.13 | - | - |

AA = African American, EA = European-American, His = Hispanic-American; ACR clinical criteria for SLE and Autoantibody prevalences are expressed as the proportion of subjects demonstrating the trait. ACR clinical criteria were complete (full data for all 11 criteria) in 49% of African-American subjects, 50% of European-American subjects, and 41% of Hispanic-American subjects.

**Supplemental Table 2** Top 50 GWAS SNPs by weighted RAS and Silhouette scores

| **SNP** | **Chro** | **Position (bp)** | **Band** | **W. RAS** | **Locus** |
| --- | --- | --- | --- | --- | --- |
| rs4632918 | 6 | 159934121 | q25.3 | 0.000022 | - |
| rs9521996 | 13 | 110380997 | q34 | 0.000042 | PARP1P1 pseudogene |
| rs863926 | 2 | 129755445 | q21.1 | 0.000052 | - |
| rs4417546 | 16 | 47789960 | q12.1 | 0.000056 | - |
| rs26725 | 5 | 106972258 | q21.3 | 0.000066 | EFNA5 |
| rs10980684 | 9 | 110836019 | q31.3 | 0.000106 | LPAR1 |
| rs6088515 | 20 | 32573703 | q11.22 | 0.000124 | DYNLRB1 |
| rs2820223 | 6 | 35117273 | p21.31 | 0.000158 | ANKS1A |
| rs16950711 | 17 | 14799633 | p12 | 0.000188 | - |
| rs10762360 | 10 | 71729512 | q22.1 | 0.000214 | LRRC20 |
| rs1535815 | 6 | 35200113 | p21.31 | 0.000262 | TCP 11 |
| rs10416963 | 19 | 17623890 | p13.11 | 0.000332 | UNC13A |
| rs12479030 | 2 | 174954179 | q31.1 | 0.000364 | OLA1 |
| rs494965 | 11 | 119558860 | q23.3 | 0.000390 | OAF homolog |
| rs10519757 | 15 | 30938702 | q13.3 | 0.000420 | FMN1 |
| rs3757319 | 6 | 152006305 | q25.1 | 0.000422 | C6orf97 |
| rs4778708 | 15 | 77649958 | q25.1 | 0.000483 | Dp-1 pseudogene |
| rs11219769 | 11 | 124125357 | q24.2 | 0.000529 | VSIG2 |
| rs847851 | 6 | 35012562 | p21.31 | 0.000547 | ANKS1A |
| rs10967250 | 9 | 26219284 | p21.2 | 0.000687 | - |
| rs354694 | 2 | 143759249 | q22.2 | 0.000697 | ARHGAP15 |
| rs17494870 | 18 | 8461372 | p11.23 | 0.000705 | PTPRM |
| rs4302457 | 4 | 10063242 | p16.1 | 0.000707 | ZNF518B |
| rs10263848 | 7 | 156221865 | q36.3 | 0.000745 | NOM1 |
| rs9963028 | 18 | 36478943 | q12.3 | 0.000775 | - |
| rs6942902 | 7 | 31110744 | p15.1 | 0.000977 | NEUROD6 |
| rs6619774 | X | 94240862 | q21.33 | 0.001275 | - |
| rs10784318 | 12 | 61567713 | q14.2 | 0.001442 | PPM1H |
| rs4394668 | 1 | 12605495 | p36.22 | 0.001448 | DHRS3 |
| rs9967792 | 2 | 191799941 | q32.3 | 0.001522 | STAT4 |
| rs2073272 | 6 | 90378676 | q15 | 0.001566 | ANKRD6 |
| rs1335130 | 9 | 31173551 | p21.1 | 0.001584 | - |
| rs1323823 | 1 | 57938688 | p32.2 | 0.001706 | DAB1 |
| rs2256229 | 20 | 1465979 | p13 | 0.001836 | SIRPD |
| rs9381493 | 6 | 47023085 | p12.3 | 0.001906 | GPR116 |
| rs753458 | 5 | 150961452 | q33.1 | 0.002022 | FAT2 |
| rs1566929 | 12 | 115346533 | q24.22 | 0.002024 | - |
| rs10171212 | 2 | 78670929 | p12 | 0.002166 | - |
| rs5769165 | 22 | 45516200 | q13.31 | 0.002381 | TBC1 |
| rs17640978 | 13 | 42632690 | q14.11 | 0.002575 | DNAJ |
| rs10876765 | 12 | 54075813 | q13.2 | 0.002969 | OR6C65 |
| rs2208858 | 6 | 18807196 | p22.3 | 0.003300 | - |
| rs7726998 | 5 | 21611296 | p14.3 | 0.003704 | CDH12 |
| rs10830794 | 11 | 91261350 | q14.3 | 0.003868 | - |
| rs11032230 | 11 | 33319442 | p13 | 0.004159 | HIPK3 |
| rs12148147 | 15 | 60550260 | q22.2 | 0.004555 | - |
| rs1736512 | 6 | 22154296 | p22.3 | 0.004773 | - |
| rs4579355 | 6 | 42193203 | p21.1 | 0.005400 | C6orf132 |
| rs358377 | 3 | 13156905 | p25.1 | 0.006095 | - |
| rs8181889 | 13 | 52511991 | q21.1 | 0.006358 | OLFM4 |

Chro = chromosome, W. RAS = weighted RAS score as outlined in the main text, Locus = the gene ID which the SNP is located within or nearby. If no genes were located within 75Kb in either direction, this was indicated with a “-“ in the Locus column. Shading is used to indicate the SNPs which were located within or near genes with immune system relevance that were then considered for replication.

**Supplemental Table 3** Rank-order statistical results for each SNP chosen from the GWAS for validation in each ancestral background separately

| **SNP** | **EA** | **AA** | **Asian** | **HA** |
| --- | --- | --- | --- | --- |
| LRRC20 rs10762360 | 448 | 6459 | 46829 | 2511 |
| PPM1H rs10784318 | 431 | 47548 | 4403 | 2090 |
| LPAR1 rs10980684 | 2935 | 860 | 48170 | 14497 |
| ANKS1A rs2820223 | 2718 | 2792 | 22216 | 5209 |
| PTPRM rs17494870 | 2010 | 57 | 794 | 22873 |
| EFNA5 rs26725 | 23070 | 3938 | 41215 | 1639 |
| VSIG2 rs11219769 | 8967 | 109663 | 5123 | 4478 |

The number in each box corresponds to the rank order for the SNP in a given ancestral background out of 500,568 SNPs, with number 1 being the most strongly associated SNP in each background.

**Supplemental Table 4** Results of individual autoantibody regressions in African-American SLE patients in the validation cohort

|  | | Ro | La | Sm | RNP | DNA | Ro with Sm | Ro with DNA |
| --- | --- | --- | --- | --- | --- | --- | --- | --- |
| LRRC20 | OR | 1.13 | **2.40** | 0.83 | 0.96 | 1.22 | - | - |
|  | p | 0.57 | **1.6x10^-4^** | 0.42 | 0.87 | 0.37 | - | - |
| PPM1H | OR | 0.88 | 0.69 | 0.95 | 0.98 | 1.33 | - | - |
|  | p | 0.55 | 0.21 | 0.8 | 0.91 | 0.19 | - | - |
| LPAR1 | OR | **1.39** | 0.77 | **1.45** | 1.06 | 1.23 | **2.19** | - |
|  | p | **0.04** | 0.32 | **0.06** | 0.78 | 0.36 | **0.004** | - |
| ANKS1A | OR | **1.95** | 0.94 | 0.8 | 1.43 | **2.12** | - | **3.14** |
|  | p | **0.09** | 0.88 | 0.55 | 0.22 | **0.03** | - | **3.5x10^-4^** |
| PTPRM | OR | 1.42 | 1.39 | 1.07 | 1.31 | 0.77 | - | - |
|  | p | 0.12 | 0.29 | 0.78 | 0.24 | 0.27 | - | - |
| EFNA5 | OR | 1.05 | 0.76 | 1.10 | **1.73** | 1.03 | - | - |
|  | p | 0.85 | 0.42 | 0.72 | **0.02** | 0.91 | - | - |
| VSIG2 | OR | 1.1 | 1.21 | 0.81 | 0.97 | 0.92 | - | - |
|  | p | 0.64 | 0.44 | 0.32 | 0.9 | 0.71 | - | - |

OR = odds ratio and p = p value for the two variables in the given regression model.

**Supplemental Table 5** Results of individual autoantibody regressions in European-American SLE patients in the validation cohort

|  | | Ro | La | Sm | RNP | DNA | RNP lacking Sm |
| --- | --- | --- | --- | --- | --- | --- | --- |
| LRRC20 | OR | 1.54 | 1.23 | 1.59 | 1.04 | 1.34 | - |
|  | p | 0.17 | 0.68 | 0.28 | 0.92 | 0.34 | - |
| PPM1H | OR | 0.71 | **4.32** | 2.15 | 1.4 | 0.69 | - |
|  | p | 0.36 | **0.004** | 0.11 | 0.46 | 0.28 | - |
| LPAR1 | OR | 0.9 | 1.15 | 1.68 | 1.64 | 1.53 | - |
|  | p | 0.76 | 0.83 | 0.28 | 0.21 | 0.19 | - |
| ANKS1A | OR | 0.84 | 1.38 | 0.89 | 0.9 | 0.86 | - |
|  | p | 0.55 | 0.53 | 0.77 | 0.78 | 0.58 | - |
| PTPRM | OR | 0.85 | 0.38 | 0.96 | 0.71 | 1.13 | - |
|  | p | 0.57 | 0.11 | 0.93 | 0.34 | 0.64 | - |
| EFNA5 | OR | 0.86 | 1.17 | 0.46 | 1.17 | 1.13 | - |
|  | p | 0.68 | 0.82 | 0.12 | 0.71 | 0.72 | - |
| VSIG2 | OR | 1.01 | 1.10 | **0.51** | **2.02** | 0.81 | **4.34** |
|  | p | 0.99 | 0.86 | **0.08** | **0.07** | 0.5 | **0.0016** |

OR = odds ratio and p = p value for the two variables in the given regression model.

**Supplemental Table 6** Results of individual autoantibody regressions in Hispanic-American SLE patients in the validation cohort

|  | | Ro | La | Sm | RNP | DNA | Ro with Sm | RNP lacking Sm |
| --- | --- | --- | --- | --- | --- | --- | --- | --- |
| LRRC20 | OR | 0.89 | 2.61 | 0.98 | 1.48 | 0.62 | - | - |
|  | p | 0.72 | 0.13 | 0.94 | 0.26 | 0.19 | - | - |
| PPM1H | OR | 1.11 | **3.77** | 0.87 | 1.03 | 0.63 | - | - |
|  | p | 0.75 | **0.02** | 0.71 | 0.94 | 0.18 | - | - |
| LPAR1 | OR | **1.83** | 0.73 | **1.72** | 1.11 | 1.56 | **2.46** | - |
|  | p | **0.08** | 0.63 | **0.10** | 0.77 | 0.24 | **0.03** | - |
| ANKS1A | OR | 0.65 | 0.35 | 1.12 | 0.86 | 0.46 | - | - |
|  | p | 0.26 | 0.16 | 0.78 | 0.67 | 0.06 | - | - |
| PTPRM | OR | 1.14 | 1.7 | 1.22 | 1.02 | 1.32 | - | - |
|  | p | 0.71 | 0.45 | 0.61 | 0.96 | 0.48 | - | - |
| EFNA5 | OR | 0.95 | 1.08 | 0.64 | 0.7 | 1.71 | - | - |
|  | p | 0.89 | 0.91 | 0.26 | 0.34 | 0.19 | - | - |
| VSIG2 | OR | 0.93 | 0.63 | **0.46** | **1.81** | 0.67 | - | **3.53** |
|  | p | 0.85 | 0.57 | **0.07** | **0.02** | 0.24 | - | **0.02** |

OR = odds ratio and p = p value for the two variables in the given regression model.

**Supplemental Table 7** Case-control analysis in African- and European-American ancestral backgrounds

| **Locus and Allele** | **African-American ancestry** | | | | **European-American ancestry** | | | |
| --- | --- | --- | --- | --- | --- | --- | --- | --- |
|  | **Cases MAF** | **Controls MAF** | **Odds Ratio**  **(95% CI)** | **p value** | **Cases MAF** | **Controls MAF** | **Odds Ratio (95% CI)** | **p value** |
| **LRRC20 rs10762360 G** | 0.324 | 0.370 | 0.81 (0.65-1.03) | 0.08 | 0.250 | 0.246 | 1.02 (0.72-1.45) | 0.92 |
| **ANKS1A rs2820223 C** | 0.119 | 0.093 | 1.33 (0.93-1.90) | 0.12 | 0.452 | 0.500 | 0.83 (0.61-1.13) | 0.23 |
| **PTPRM rs17494870 C** | 0.733* | 0.747* | 0.93 (0.72-1.20) | 0.56 | 0.420 | 0.491 | 0.75 (0.55-1.02) | 0.06 |
| **LPAR1 rs10980684 C** | 0.314 | 0.312 | 1.01 (0.80-1.28) | 0.92 | 0.307 | 0.332 | 0.89 (0.64-1.23) | 0.47 |
| **VSIG2 rs112219769 G** | 0.310 | 0.321 | 0.95 (0.75-1.21) | 0.69 | 0.281 | 0.274 | 1.04 (0.74-1.45) | 0.84 |
| **PPM1H rs 10784318 C** | 0.311 | 0.302 | 1.04 (0.82-1.32) | 0.74 | 0.371 | 0.336 | 1.16 (0.85-1.60) | 0.35 |
| **EFNA5 rs26725 G** | 0.189 | 0.188 | 1.01 (0.76-1.3) | 0.97 | 0.216 | 0.153 | 1.54 (1.04-2.29) | 0.03 |

Allele frequencies were compared between cases and controls using a chi-square test of independence. MAF = minor allele frequency, 95% CI = 95% confidence interval, * = the C allele of the PTPRM rs17494870 SNP is the minor allele in European-American subjects but is the major allele in African-Americans, and the frequency of the C allele is indicated in the MAF column for both ancestral backgrounds

**Supplemental Figure Legends**

**Supplemental Figure 1** Serum IFN-α in SLE patients stratified by PTPRM genotype in African-American (A.) and joint analysis of European- and Hispanic-American subjects (B.). Y-axis shows the serum IFN-α activity score as outlined in the Methods section. Bars show the median, error bars show the interquartile range. P-values calculated using the Mann-Whitney U test for a difference between the two homozygous genotype groups.

**Supplemental Figure 2** Serum IFN-α in SLE patients stratified by LRRC20 genotype in African-American (A.) and joint analysis of European- and Hispanic-American subjects (B.). Y-axis shows the serum IFN-α activity score as outlined in the Methods section. Bars show the median, error bars show the interquartile range. P-values calculated using the Mann-Whitney U test for a difference between the two homozygous genotype groups.

**Supplemental Figure 1**

**
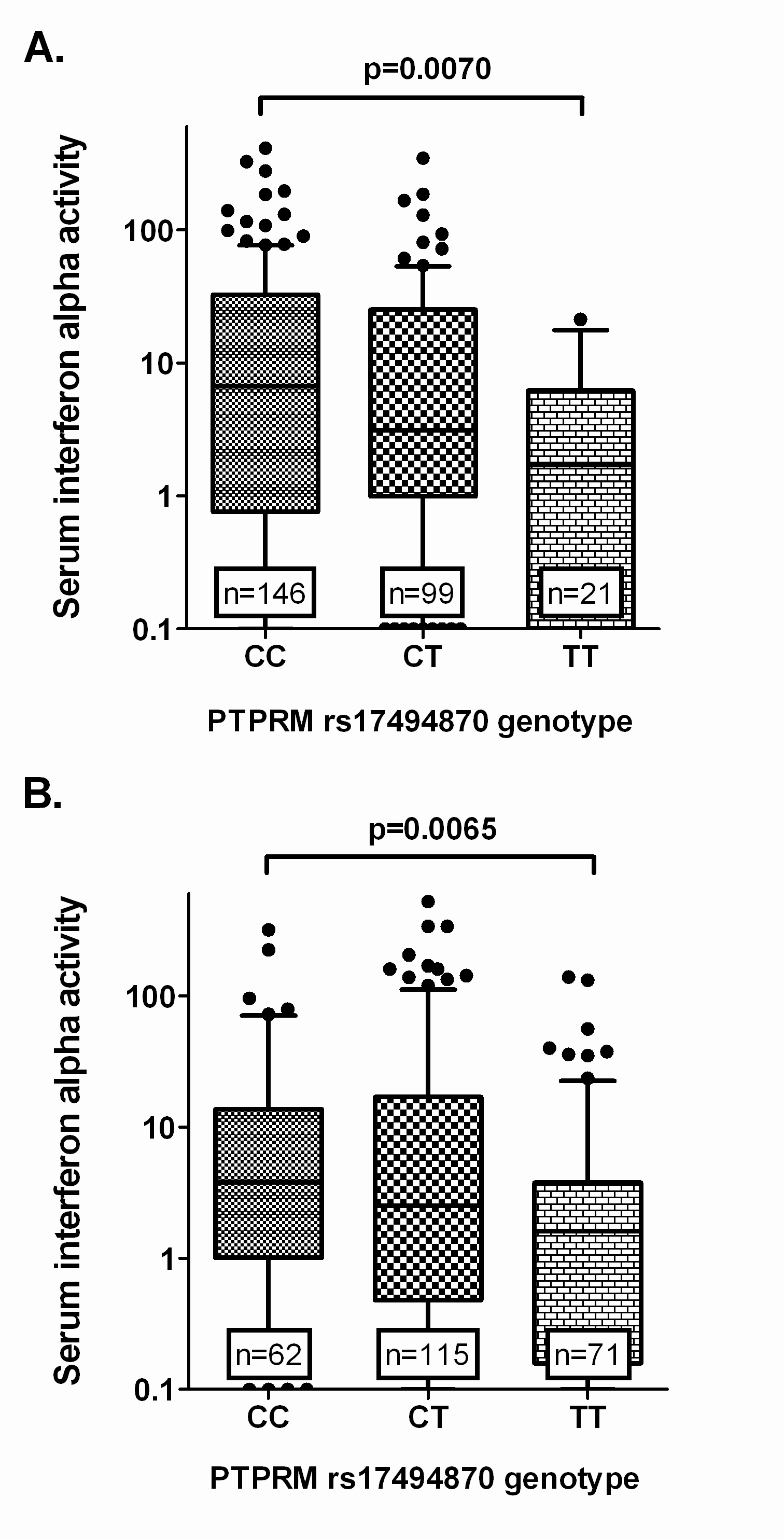
**

**Supplemental Figure 2**

**
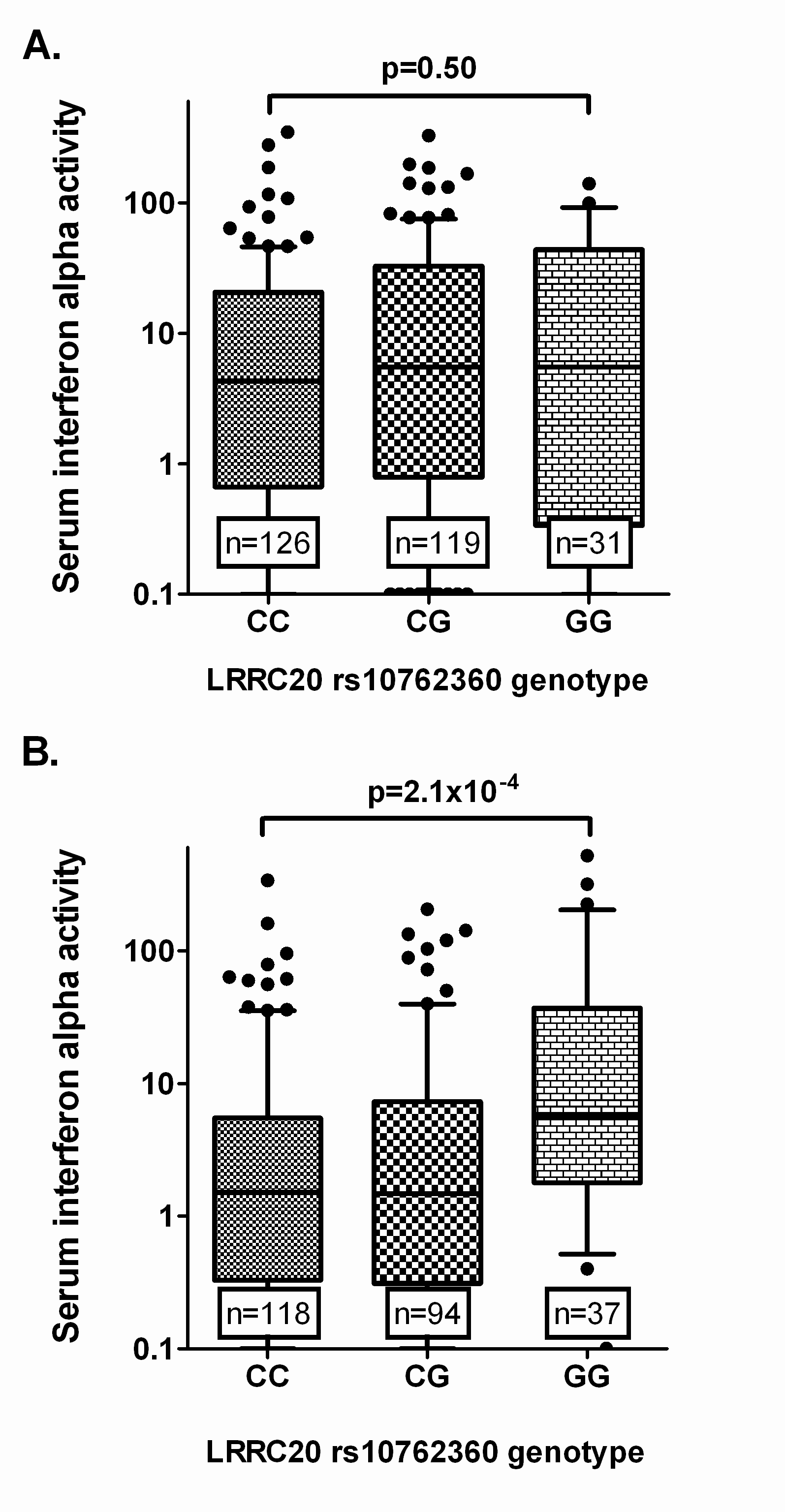
**
